# A Simple Method for Triple Stable Isotope Analysis of Cellulose, Sugar, and Bulk Organic Matter—Advances and Limitations

**DOI:** 10.1002/rcm.9957

**Published:** 2024-12-02

**Authors:** Matthias Saurer, Manuela Oettli, Marco M. Lehmann

**Affiliations:** ^1^ Swiss Federal Institute for Forest, Snow and Landscape Research WSL Birmensdorf Switzerland

**Keywords:** carbon, deuterium, isotope ratios, method development, oxygen

## Abstract

**Rationale:**

Determining several isotope ratios in one analysis multiplies the information that can be retrieved from a sample in a cost‐efficient way. The stable isotope ratios of hydrogen (δ^2^H), carbon (δ^13^C), and oxygen (δ^18^O) in organic compounds are highly relevant due to their complimentary hydroclimatic and physiological signals. Different types of organic material reflect different processes and integration times, like short term in leaf sugars and long term in tree ring cellulose, but currently, no simple method exists for their triple isotope analysis.

**Methods:**

Here, we present a method that enables the isotopic analyses of the three elements H, C, and O in one run and is applicable to different types of carbohydrates and bulk organic matter. We discuss all steps required from water vapor equilibration necessary for obtaining reliable δ^2^H values of carbon‐bound H to high‐temperature conversion (HTC) of the sample to CO and H_2_ and to the mass‐spectrometric isotope‐ratio analysis.

**Results:**

We show that reliable triple isotope analysis is possible for a large range of samples, although it results in some reduction of precision compared to individual isotope analysis. Important considerations are the equilibration procedure, the type of autosampler, selection of HTC reactor, the influence of nitrogen in the sample, the verification of δ^13^C values obtained by HTC versus combustion, and the selection of reference materials.

**Conclusions:**

By presenting a relatively simple triple‐isotope method, we promote the use of multi‐isotope studies in environmental sciences, which helps in addressing many important climate and ecological research challenges that we face today.

## 1 Introduction

Stable isotope analysis of plant compounds is widely used in climate and environmental sciences [1]. Most studies so far involving carbohydrates focused on carbon and oxygen isotope ratios (δ^13^C and δ^18^O), whereas only recently, there was an increasing number of studies investigating hydrogen isotope ratios (δ^2^H) [2, 3]. It is well established that the combination of different isotope ratios from the same sample yields additional insights into plant functioning. This has often been applied concerning carbon and oxygen [4, 5], but much less studies involved also hydrogen and thus applied a triple isotope approach [6, 7]. It would, however, be very valuable to include hydrogen to the more commonly used carbon and oxygen isotopes, not only for cellulose but also for sugars, and sugars derived from the digestion of starch and organic matter in general. This will be helpful for addressing many environmental and climate questions and for better deciphering the isotope fractionations occurring within plants [8, 9, 10]. For more widespread application of the triple isotope approach and for cost‐efficiency, a method analyzing “all in one” would be very valuable.

The principal idea for achieving a triple ^2^H/^1^H‐^13^C/^12^C‐^18^O/^16^O isotope analysis (from now on, triple isotope analysis) is converting the organic sample to H_2_ and CO in a high‐temperature conversion (HTC) process, sometimes called pyrolysis. The two gasses can be separated by gas chromatography (GC) or by trapping and releasing CO, with subsequent analysis of δ^2^H on H_2_ and δ^13^C and δ^18^O analysis on CO with an isotope‐ratio mass spectrometer (IRMS). Although the “gold‐standard” method for analysis of δ^13^C is via combustion resulting in CO_2_ [11], it has been shown that reliable δ^13^C analysis is possible from HTC considering appropriate corrections and calibrations [12, 13]. The analysis of H_2_ is in principle straight forward, as modern IRMS allows jumping from one analyzed gas to another within one run (e.g., from H_2_ to CO). There is, however, the requirement of controlling the exchangeable OH groups before δ^2^H analysis as oxygen‐bound hydrogen atoms exchange with hydrogen atoms of the surrounding liquid water and water vapor. This is usually done by equilibration of the samples with water of known isotopic composition [14, 15]. Only the carbon‐bound H atoms contain the relevant and permanently stored climate and environmental information. Therefore, reliable triple isotope analysis also requires including a prior equilibration step. We rely here on a high‐temperature (130°C) equilibration method that is applicable also for soluble materials, like sugars [16]. A method for triple isotope analysis with equilibration of OH‐groups was previously published, but the method works only for cellulose, and it involves a rather complex setup for the ^2^H equilibration that may not be easy to replicate and might not be usable on all HTC devices [17]. Therefore, there is currently a lack of a simple and robust method.

To be widely applicable, an analysis method should ideally be suited for different types of materials, like cellulose (often used in tree ring studies), sugars (for studies involving process‐based and postphotosynthetic investigations), and bulk organic samples like leaves and soil as used for many ecological and soil–plant interaction studies. We investigate here the requirements for successful triple isotope analysis of various materials, including also the analysis of the nitrogen‐containing substances that need consideration for the thermal decomposition and the type of HTC reactor due to a possible interference in the analysis of δ^2^H by formation of hydrogen cyanide [18]. We describe all relevant procedures as is important for obtaining comparable results among laboratories [19]. Accordingly, we (1) present a simple triple‐isotope method expanding on an equilibration technique recently published for hydrogen only [16], (2) compare the precision and accuracy of this method to individual isotope measurements, (3) compare results of nitrogen‐containing materials in different HTC reactors (chromium vs. glassy carbon), and (4) investigate the influence of different autosampler types.

## 2 Methods

### 2.1 Water Vapor Equilibration

The first step in the analysis chain is the equilibration with water vapor. To determine or eliminate the influence of exchangeable OH‐groups on the δ^2^H‐values of carbohydrates, two principal methods have been applied: either nitration [20] or equilibration with water(s) of known isotopic composition [14]. The first may be too complicated and hazardous for routine use but can still be useful for preparation of standard materials [16]. For the second approach, different protocols have been suggested for equilibration (not considering analysis of unequilibrated samples as is also sometimes done), ranging from room temperature equilibration for extended periods of time and requiring no special equipment [21] to more sophisticated setups, usually at higher temperatures [15, 22, 23]. This situation may not be ideal as various methods are applied, potentially impacting the actual degree of exchange of oxygen‐bound H and interlaboratory isotope precision. A table listing the different δ^2^H methods has recently been published [24]. Here, we adopt and promote the use of a high‐temperature (130°C) method recently published because this temperature is required for water vapor equilibration of sugars and because the setup is relatively simple with equipment mostly available commercially and therefore possible to replicate by other laboratories.

#### 2.1.1 Equilibration Device

**FIGURE 1:**
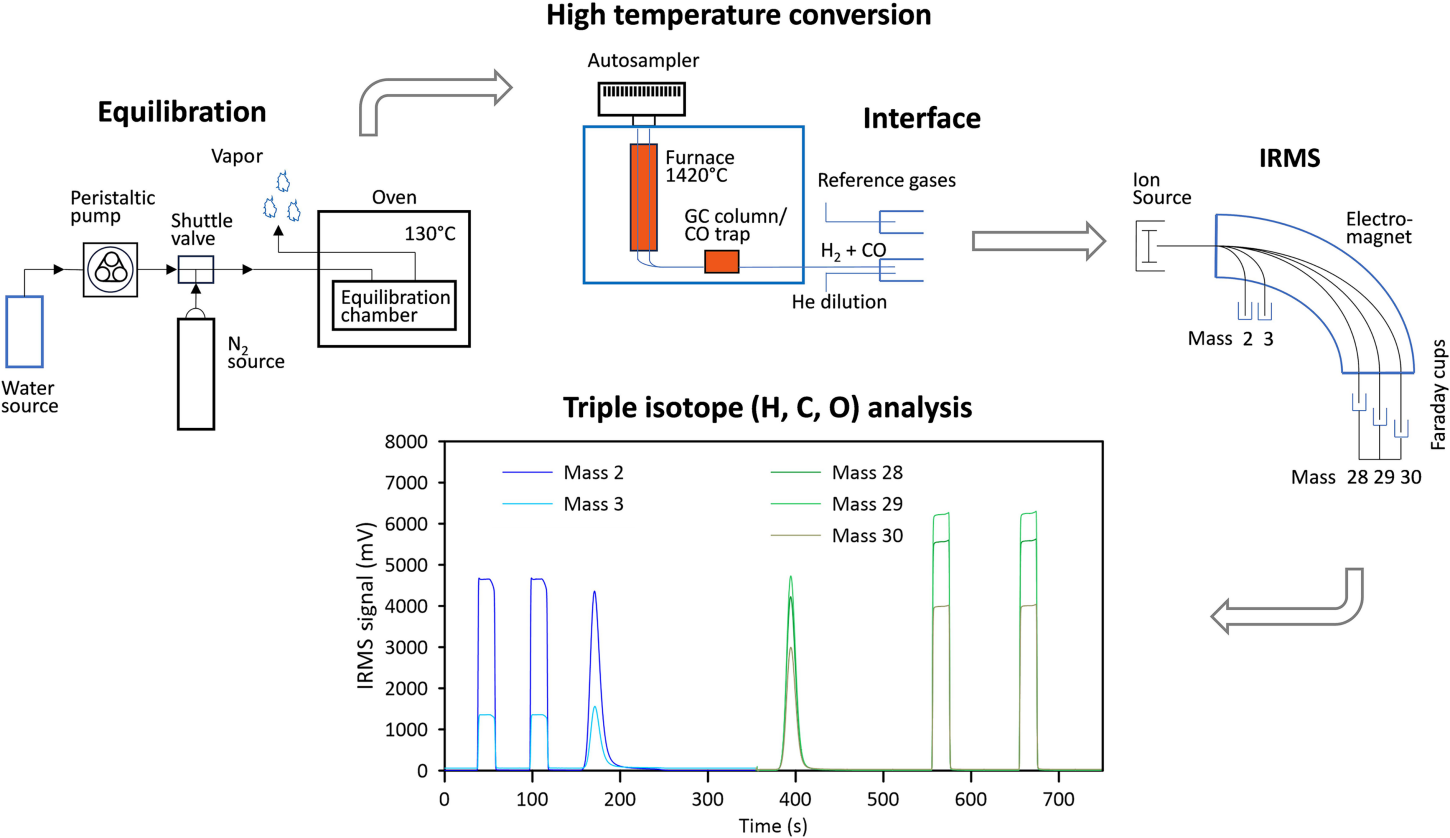
Triple isotope setup, including water vapor equilibration, high‐temperature conversion, interface, IRMS, and analysis showing an example IRMS chromatogram of a triple isotope analysis.

The equilibration system consists of a heating oven (Binder, Tuttlingen, Germany), a simple, in‐house designed equilibration chamber located in the oven and a peristaltic pump (Gilson Incorporated, Middleton, USA) placed outside the oven. The equilibration chamber is a cubic stainless‐steel chamber of dimensions 178 × 178 × 32 mm covered with a stainless‐steel metal plate using one stainless steel clamp at each corner and sealed with a heat‐stable Viton® O‐rings (Maagtechnic AG, Dübendorf, Switzerland, Prod. No. 15087359) located in a groove (Figures S1 and S2). The dimensions of the chamber are such as to hold a sampler carousel (Zero Blank Autosampler, N.C. Technologies S.r.l., Milano, Italy) for solid samples with 50 or 100 positions for samples and reference materials packed in silver capsules. This chamber is the only part that is custom made and may be produced, for example, in a university workshop. The chamber cover has inlet and outlet lines (stainless steel, 1/16 in.), where the inlet is leading out of the oven to the peristaltic pump via connection to a santoprene pump tubing. The beginning line of the santoprene pump tubing is inserted into a 50‐mL falcon tube containing the equilibration water located outside the oven. The outlet line from the equilibration chamber is vent to the laboratory atmosphere or inserted into an open glass vessel where the vapor condenses and enables to check the amount of water (Figure 1).

#### 2.1.2 Equilibration Procedure

The oven temperature is set to 130°C, ensuring immediate evaporation of water after entering the equilibration chamber. The peristaltic pump provides a constant flow of the equilibration water of 1.7 mL/h into the equilibration chamber. After 2 h of equilibration, the feeding capillary is switched to a line delivering dry nitrogen gas (N_2_ 5.0, PanGas AG, Dagmersellen, Switzerland) with a pressure of one bar for 2 h to ensure drying the samples, that is, achieve a complete removal of gaseous water in the chamber, which is still kept at 130°C [16].

### 2.2 Isotope Analysis

#### 2.2.1 Autosamplers

After equilibration, the samples (still hot) are immediately transferred to the autosampler on the instrument used for analysis. It is important to transfer the samples as fast as possible to avoid any isotopic reequilibration of the sample with air moisture and water absorption. Different types of autosamplers are used by different manufacturers, where samples are often kept in helium or argon, but sometimes only covered by a simple lid and therefore exposed to atmospheric air, including moisture (only in the last step before analysis, samples will be flushed with helium). Although this is not a concern for most types of isotope analysis, it is important to consider for hydrogen isotope analysis due to potential exchange of oxygen‐bound hydrogen and the fact that samples may be “waiting” in the autosampler tray for the analysis for several hours. In our study, (1) we used Zero Blank Autosampler (N.C. Technologies S.r.l., Milano, Italy). Here, the autosampler carousel was evacuated to 0.01 mbar after placing the samples and afterwards filled with dry helium gas to 1.5 bar to avoid any contact with ambient vapor (Figure S3). And (2) we also used Autosampler on the Pyrocube‐instrument (Elementar, Hanau, Germany). The samples on this tray are covered by a simple plastic cover and therefore in air, but the tray can be heated to 80°C to reduce adsorption of moisture. Additionally, we added a custom‐made hood of plexiglass acrylic that can be flushed with Argon to minimize contact of sample with air moisture (Figure S4).

#### 2.2.2 Thermal Decomposition and Reactor Tubes

The devices for decomposition of the samples to H_2_ and CO consist of an oven with a reactor‐tube capable of holding 1420°C and a separation module for the two gasses to be analyzed (either GC‐column or CO‐trap). We used (1) Thermal‐conversion Elemental Analyser TC/EA (Thermo, Bremen, Germany) with a single ceramic tube, filled either with glassy carbon or with chromium, and a GC‐separation column (5A mol sieve) and (2) Pyrocube (Elementar, Hanau, Germany) equipped with a tube‐in‐tube reactor setup (outer tube ceramic, inner tube glassy carbon) and a CO‐trap that collects the CO at 40°C and releases it at 150°C. The type of reactor is an important consideration because on the one hand, the δ^18^O analysis (and thus triple isotope analysis) is only possible with an inner glassy carbon tube, preventing oxygen isotope exchange with the hot walls of the ceramic tube (Al_2_O_3_), although this is not needed for δ^2^H analysis only. On the other hand, the filling of the reactor with chromium may be useful for δ^2^H analysis of nitrogen‐containing compounds, but this would prevent δ^13^C and δ^18^O analysis on CO.

#### 2.2.3 Interface and IRMS

The interface provides the link to the IRMS and a dilution of the sample gas appropriate for the isotope analysis and also is capable of adding reference gasses (H_2_ and CO). We used ConFlo III and Conflo IV interfaces (Thermo, Bremen, Germany). The latter has the advantage that it enables change in the dilution during an analysis of a sample (when changing from H_2_ to CO analysis, i.e., applying a “peak jump”), which is useful for adapting the gas‐sample signal sensitivity for triple isotope analysis. We were using as IRMS a DeltaPlus XP and a MAT253 (Thermo, Bremen, Germany). ^2^H/^1^H is derived from ^2^H^1^H/^1^H^1^H analysis and ^18^O/^16^O and ^13^C/^12^C from ^12^C^18^O/^12^C^16^O and ^13^C^16^O/^12^C^16^O analysis, respectively. All isotope delta values (δ) are calculated as follows:

(1) $$\delta =\frac{R_{\text{Sample}}-R_{\text{Standard}}}{R_{\text{Standard}}},$$

where *R* is ^2^H/^1^H, ^18^O/^16^O, or ^13^C/^12^C of the sample (*R*_*Sample*_) and standard (*R*_*Standard*_). *R*_*Standard*_ is used for the normalization of *R*_*Sample*_ to the international scale, which is Vienna Standard Mean Ocean Water (VSMOW) for δ^18^O and δ^2^H and Vienna Pee Dee Belemnite (VPDB) for δ^13^C. To express the resulting δ values in permil (‰), results have to be multiplied by 1000. Besides the isotope ratios, the IRMS analysis also enables calculation of the total element concentrations in % based on the mass‐spectrometer peak areas.

#### 2.2.4 Overall Configuration

We mainly used the two following setups based on the instruments described above:

1. Setup “HTC single” for δ^2^H analysis only: zero‐blank autosampler—TC/EA with ceramic tube and either chromium or glassy carbon filling—Conflo III—IRMS DeltaPlus XP.
2. Setup “HTC triple” for triple isotope analysis: Elementar autosampler with custom‐made cover—Pyrocube with glassy‐carbon tube in a ceramic tube with glassy‐carbon filling—Conflo IV—IRMS MAT253.

The overall analysis scheme for the setup “HTC triple” is depicted in Figure 1, with additional information provided in Figures S1–S4.

For δ^13^C analysis via combustion, an elemental analyzer was used (isoEArth, Sercon, Crewe, UK) in combination with IRMS (HS2022, Sercon, Crewe, UK).

### 2.3 Materials

As primary reference materials for δ^2^H, we used IAEA‐CH‐7 polyethylene foil (International Atomic Energy Agency, Vienna, Austria) for a first offset correction and USGS62, USGS63, and USGS64 caffeine standards (United States Geological Survey, Reston, Virginia, USA) [25] for the normalization using the “setup single” with the chromium reactor. These setup and standard materials were used to calibrate additional in‐house reference materials for δ^2^H, in particular cellulose and sugars for use in routine analyses and on all systems (including “setup triple”). We made also comparison of these standards measured with water vapor equilibration with the same standards as nitrated cellulose [16]. We used both commercially available (IAEA‐CH‐3, cellulose Merck, cellulose Fluka) and in‐house produced cellulose (“Spruce,” “Beech,” “Spain,” and “Siberia”). In‐house produced standards are useful for the identical‐treatment protocol compared to samples. In particular, the proportion of exchangeable H of commercial cellulose may differ from inhouse‐produced cellulose due to the different microscopical structure. Cellulose was extracted according to [6], modified by [16].

Additional standard materials were selected based on their isotope range not only concerning δ^2^H but also δ^18^O and δ^13^C. These included stem and twig wood organic matter and cellulose from a Siberian and Swiss *Larix* tree. For sugars, we used commercial ones (Sucrose: Merck, Prod. No. 1.07687; household sugars “Finish sucrose,” Suomalainen Taloussokeri, Kantvik, Finland; “Russian sucrose,” household sugar from a Russian supermarket supplier).

For testing the influence of nitrogen on δ^2^H‐results, we used the following materials: B2164 (homogeneous batch of Algae, Bladderwrack) provided by the National Institute of Standards and Technology (NIST, Maryland, USA) with a relatively low C content of 32.83%, H‐content of 4.96%, and N content of 1.26% but high sulfur content of 2.289% (normal leaves < 0.5%); Peach Leaves material (NIST 1547); and inhouse produced “Beech wood,” “Fungi,” and “Insect.” These homogenized materials cover a range of N content from 0.2% to 10.4%.

#### 2.3.1 Preparation of Samples and Standards

All reference materials were oven dried at 60°C for 48 h and stored in a desiccator at low relative humidity (2%–5%) until further use. For the analysis, 1 mg of material was packed into 3.3 × 5 mm silver foil capsules (IVA, Prod. No. SA76980506). For sugars, an equivalent amount pipetted as solution in a capsule, freeze dried, and afterwards additionally packed in a second capsule of the same size and folded again [16]. The reason for the double packing was the observation the liquified sugar samples might leak out of single‐packed capsules during the hot water vapor equilibration, which could lead to a loss of sample. The double packing did not have a negative impact on the equilibration itself, as indicated by the high and consistent degree of calculated proportion of exchange for sugars [16].

### 2.4 Calculations and Calibration

A two‐point calibration was applied for all isotope ratios using inhouse‐produced reference materials with different isotope ratios that were previously normalized to the international VSMOW and VPDB scales using IAEA materials. Additional corrections for memory effect or instrument drift were made if necessary. The %‐proportion of exchanged hydrogen (*x*_*e*_) was calculated based on two equilibrations using two isotopically distance waters as follows:

(2) $$x_{e}=\frac{\delta^{2}H_{e1}-\delta^{2}H_{e2}}{\alpha_{e-w}\;\left(\delta^{2}H_{w1}-\delta^{2}H_{w2}\right)},$$

where δ^2^H_e1_ and δ^2^H_e2_ are the measured δ^2^H values of the two equilibrated samples, δ^2^H_w1_ and δ^2^H_w2_ are the δ^2^H values of the two waters used, and α_*e‐w*_ is the isotope fractionation factor of 1.082 for cellulose [22]. In principle, α_*e‐w*_ needs to be adapted for different compounds and molecules with different functional groups [14]. We consider α_*e‐w*_ of cellulose to be the same for other carbohydrates, as they all have the exchangeable hydrogen on hydroxyl groups. The isotope fractionation factor we use is in the range proposed in other studies [14, 15].

Once *x*_*e*_ is determined, the carbon‐bound, nonexchangeable isotope ratio δ^2^H_ne_ can be calculated as follows:

(3) $$\delta^{2}H_{\mathrm{ne}}=\frac{\delta^{2}H_{e1}-x_{e}\;\alpha_{e-w}\;\delta^{2}H_{w1}-1000\;x_{e}\;\left(\alpha_{e-w}-1\right)}{1-x_{e}}$$

using one of the two equilibrations (in this example, equilibration with Water 1; δ^2^H_e1_ and δ^2^H_w1_). This equation can also be used to calculate δ^2^H_ne_ when performing only one equilibration and assuming *x*_*e*_ to be known (i.e., when the same kind of samples is analyzed, like tree‐ring cellulose samples).

## 3 Results and Discussion

### 3.1 Triple Isotope Analysis

**FIGURE 2:**
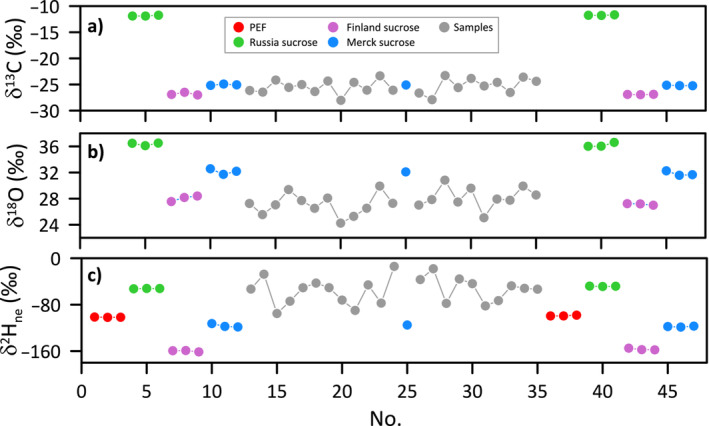
Typical sequence with standard materials and samples for triple isotope analysis of sugars (samples extracted and purified from leaves) showing calibrated δ^13^C, δ^18^O, and δ^2^H_ne_ values, as obtained after equilibration with a water of known isotopic composition.

We show as an example the isotope results of a series of sugar samples together with the relevant standards analyzed with the setup “HTC‐triple” (Figure 2). After water vapor equilibration, drying and placing the samples in the autosampler, the analysis is started. First, δ^2^H is analyzed on H_2_ and then δ^13^C and δ^18^O on CO. The trapping of CO and subsequent release by temperature increase of the respective trap allows for sufficient time between the H_2_ and CO and provides an optimal (symmetrical) peak shape (Figure 1), unlike in GC‐separation in many TC/EA systems that often results in peak tailing [26]. Peak areas also allow amount %‐content calculations relative to total amount, which is important mainly for quality control (e.g., to detect weighing mistakes or loss of sugars). A sequence typically consists of a standard block at the beginning, comprising several standards that cover a wide range of isotope values for all three isotopes, including a quality control standard (Merck sucrose) that is not used during calibration, followed by the samples (with a quality control every ~12 samples), and ending with a standard block similar as at the beginning (Figure 2). Standard and samples are of similar composition and structure as far as possible to control possible variations in the equilibration process (with the exception of PEF, a material that does not exchange any H during equilibration, i.e., *x*_*e*_ = 0). Correction for the H exchange is done according to Equation (3), providing the final δ^2^H_ne_ values.

The precision of the triple isotope measurement based on the values of the quality control is approximately ± 3.0‰ for hydrogen, ± 0.2‰ for carbon, and ± 0.3‰ for oxygen (Table 1), which is similar to the values reported for a previously published triple‐isotope method [17]. A memory correction is routinely applied by considering the difference between the actual and previous isotope values, multiplied by the “memory effect” in %, usually in the order of 1%–3% for all isotope ratios. The latter number is determined iteratively to yield the lowest possible standard deviations for the reference materials. The precision is similar for sugar and cellulose analysis. For δ^13^C, correction for the influence of glassy carbon needs to be considered by applying a two‐point calibration with standards of known values (see Section 3.4 for details). δ^18^O values should in principle not be affected by the equilibration procedure, as only the H of OH groups is exchanging with the water vapor and not oxygen. We observed, however, a slightly higher standard variation for the long‐term quality control analysis of δ^18^O of cellulose subjected to the equilibration procedure (0.3‰) compared to values without equilibration (0.2‰). The complete drying of samples is obviously important as any traces of water left on samples would affect the results [27]. For sugars, sometimes, unexpected δ^18^O changes during wetting‐drying test cycles have been reported [28]. We did not observe this for our triple‐isotope protocol but consider it important to use sugar standard materials for analysis of sugars.

**TABLE 1:** Isotope precision determined as standard deviation of repeated analysis of quality control materials (cellulose and sugar) for different techniques.

Technique	δ^13^C (‰)	δ^18^O (‰)	δ^2^H (‰)
Elemental analyzer, combustion	0.1	—	—
HTC‐single, 2 equilibrations	—	—	2.0
HTC, no equilibration	—	0.2	—
HTC‐triple, 1 equilibration	0.2	0.3	3.0

**FIGURE 3:**
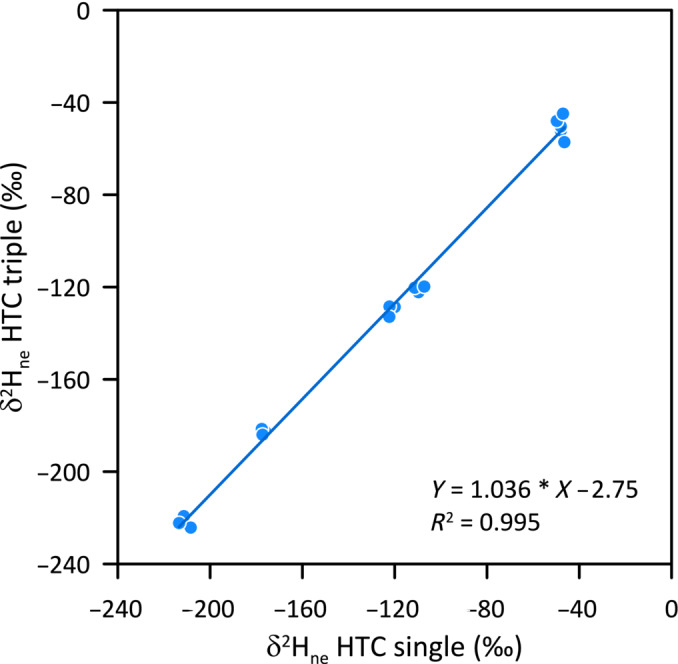
Comparison of δ^2^H_ne_ values of a range of wood and cellulose samples measured by setup “HTC single” (with two equilibrations) and setup “HTC triple” (with one equilibration). For triple analysis, a constant *x*_*e*_ of 0.15 and 0.2 was assumed for wood and cellulose, respectively.

To improve the precision for δ^2^H_ne_, such a triple isotope analysis can be done in duplicate with two different equilibration waters (which allows calculation of *x*_*e*_ of each sample). However, the elegance of the triple isotope method certainly lies in the idea of getting all this information in just one run. Comparison of δ^2^H_ne_ results obtained via triple‐isotope analysis with one equilibration and the more precise HTC‐single setup with two equilibrations provided good agreement (Figure 3). When samples of a certain type are analyzed (e.g., annual tree ring cellulose samples, all prepared and extracted in the same way), it can be safely assumed that *x*_*e*_ for all samples is very similar. Cellulose samples typically have *x*_*e*_ = 0.2 ± 0.02, wood samples *x*_*e*_ = 0.15 ± 0.02, and sugar sample *x*_*e*_ = 0.36 ± 0.02 [16]. Rather, changes in calculated *x*_*e*_ may be due to small variations in the equilibration conditions between runs (temperature, water flux, drying, etc.) rather than the sample identity. This can be considered by the analysis of similar type of standards and samples in a sequence, which therefore experience exactly the same “equilibration history.” We can assume based on extensive tests with different equilibration conditions that the equilibration is complete in our setup although for cellulose, for example, not the theoretical value of *x*_*e*_ = 0.3 is obtained. Lower values of *x*_*e*_ are expected due to intermolecular hydrogen bridges [29]. Again, the principal of identical treatment for standards and cellulose is crucial. Therefore, we recommend using working standards that have been prepared with the same method as the samples.

In contrast to the precision, the accuracy for δ^2^H_ne_ is rather difficult to assess due to the lack of comparable reference materials. There is clearly a need for interlaboratory comparisons of materials with exchangeable OH groups. With the Schuler method (2022) [16], developed at WSL and also applied here, we took several precautions to deal with this issue. Importantly, we compared δ^2^H results of carbon‐bound H of cellulose applying the nitration method (that replaces exchangeable OH‐groups with nitrate groups) and results obtained by water vapor equilibration (two waters with distinct isotope ratios) and subsequent corrections, thus using two completely independent protocols. We further compared these values with analyses done at another laboratory (University of Basel) where a rather different equilibration technique was applied (different temperature, and different apparatus). Still, we observed a very high correlation between results obtained in the two laboratories, with *r* ^2^ = 0.97 for cellulose extracted from different wood tissues, albeit with an offset of 7.1‰ [30].

### 3.2 Comparison of δ^13^C Using HTC or Combustion

The most solid and established method to determine δ^13^C of any organic matter is by combustion in an elemental analyzer with subsequent isotope analysis of CO_2_. By adding oxygen in this process and the presence of combustion catalysts in the combustion reactor, a conversion yield of 100% carbon of the sample to CO_2_ is guaranteed. In contrast, during high‐temperature conversion (HTC) at temperatures > 1400°C, without adding oxygen, organic matter is converted to CO instead. The carbon of CO is not only originating from the sample but may contain a small fraction from the reactor (glassy carbon) or reactor filling (glassy carbon as well). Conversely, there might be an isotope fractionation when carbon from the sample is deposited in the reactor rather than converted to CO. This deposition might depend on the C:O ratio of the sample. This needs thorough evaluation and appropriate corrections, as detailed below. Although δ^13^C analysis by HTC has been done before by other laboratories [12, 13] as well as our own with the equipment as described here [31], we repeated this comparison, as it is an integral part of the triple isotope analysis and still not widely used.

**FIGURE 4:**
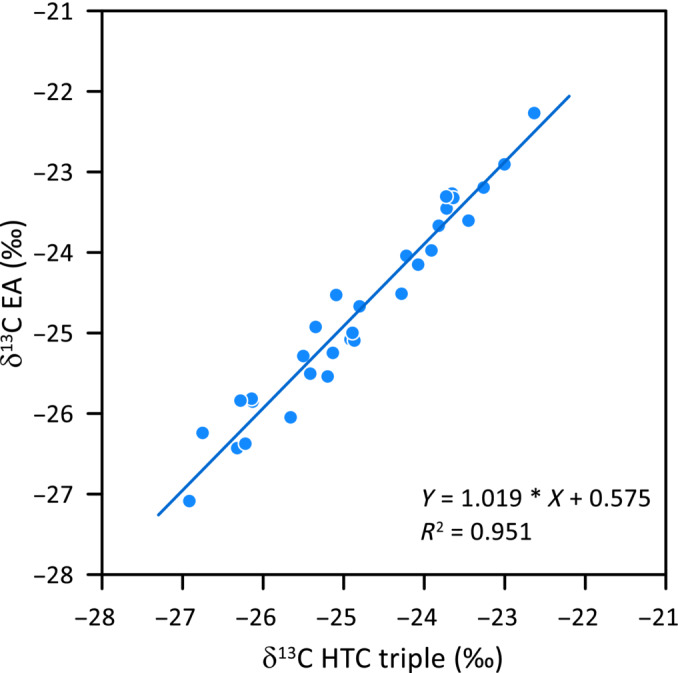
Comparison of δ^13^C values of a range of cellulose samples determined by combustion in an elemental analyzer (EA) and by the setup “HTC triple.”

A correction for the carbon isotope fractionation during HTC can be done either by reanalyzing a part of the samples by combustion [13] or by analysis of standard materials similar in type to the samples within the same run. In both cases, a linear correction is obtained where the dampening of ~15% is corrected for. The underlying assumption, which seems to be well fulfilled, is that the amount of carbon from the reactor contributing to CO is constant. We routinely applied a two‐point calibration for each run based on materials with known δ^13^C values to correct for the addition of ~15% of carbon from reactor and reactor filling. The such calibrated results show that a high correlation between δ^13^C values obtained by HTC and by combustion, with a slope of the correlation line close to 1 (Figure 4). The average difference between δ^13^C values measured by the two methods was 0.09‰ for this example. The long‐term standard deviation of reference materials is ~0.2‰ (Table 1).

### 3.3 δ^2^H Analysis of N‐Bearing Compounds

An essential requirement for triple isotope analysis is that a reactor based on glassy carbon tube and filling may be used. However, previous studies showed that N‐bearing organic materials and compounds analyzed in a pure glassy carbon tube may hamper δ^2^H values because of hydrogen isotope fractionations related to the formation of alternative products, for example, hydrogen cyanid (HCN) during pyrolysis. Therefore, not all H of the organic material might be converted to H_2_ [18]. The addition of chromium to the reactor can be a solution for this issue, as the chromium scavenges all reactive elements except hydrogen and thus eliminate hydrogen isotope fractionations [18, 32]. This is particularly critical when analyzing N‐containing organic materials with a high N‐content like amino acids or caffein. However, as oxygen of the sample is trapped in a chromium‐filled reactor due to formation of chromium oxide (Cr_2_O_3_), the lack of CO hinders obviously the analysis of δ^18^O (or δ^13^C) with such a reactor, and thus, triple isotope analysis is not possible.

We therefore compared δ^2^H_ne_ values of a range of ecological relevant samples with different N content ranging from 0.2% to 10.4% with a chromium and a glassy carbon filled reactor. We tested this on our “HTC single” setup with two water vapor equilibrations for highest precision. The results showed good consistency between results obtained with the two different HTC reactors for wood, leaves, and fungi material with discrepancies below 2‰ (Table 2). The calculated *x*_*e*_ was also similar, although this may be less surprising, as *x*_*e*_ mainly depends on the offline equilibration with water vapor and not the HTC step (but there might be an influence of the autosampler type, and time the sample spends there; see below). The total‐% H was also consistent between reactors suggesting full conversion of sample H to H_2_ (data not shown). The δ^2^H_ne_ comparison was less good for algae material, although this material did not have a high %N. We therefore assume that other properties, like the relatively high sulfur content (2.3%) of this material, may have had an influence on the δ^2^H analysis, for example, by formation of H_2_S. Further, a relatively high difference was observed for the insect material with the highest %N. When plotting the N‐content of the materials versus the difference in δ^2^H_ne_ between HTC reactors, there was a moderate positive correlation (*y* = 0.62*x* − 3.04; *R* ^2^ = 0.58), consistent with results reported earlier on the influence of N on δ^2^H [18].

**TABLE 2:** Comparison of δ^2^H_ne_ values determined with two different HTC‐reactors for materials differing in N content.

Sample material	δ^2^H_ne_ Carbon (‰)	SD (‰)	δ^2^H_ne_ Chromium (‰)	SD (‰)	*x*_*e*_ Carbon (‰)	SD (‰)	*x*_*e*_ Chromium (‰)	SD (‰)	N (%)	Diff (‰)
Beech wood	−118.1	1.7	−117.5	2.0	0.15	0.01	0.12	0.03	0.20	−0.6
Algae	−112.7	0.7	−107.5	2.6	0.26	0.01	0.27	0.02	1.27	−5.2
Leaves	−107.5	3.7	−107.8	2.5	0.19	0.01	0.19	0.01	2.94	0.3
Fungus	−27.2	1.4	−25.7	1.5	0.29	0.01	0.27	0.01	4.67	−1.5
Insect	−121.3	1.4	−125.2	1.5	0.17	0.01	0.14	0.01	10.42	3.9

Abbreviations: carbon, reactor filled with glassy carbon; chromium, reactor filled with chromium; diff, difference between δ^2^H_ne_ values determined with the two reactors; SD, standard deviation; N, N content of the sample material; Xe, fraction of H exchange.

Overall, we conclude that for many important ecological materials, particularly for foliar and wood organic matter, the effect of hydrogen cyanide formation is negligible and that a reliable δ^2^H_ne_ analysis is possible with a glassy carbon reactor. However, in cases of unknown or newly applied material, we recommend comparing the samples on a system with a glassy carbon to a chromium reactor for potential isotope fractionation. Although our results are based only on the set‐up “HTC single,” we assume that our finding is applicable for the setup “HTC triple,” which also uses a glassy carbon reactor.

### 3.4 Autosampler Influence

**FIGURE 5:**
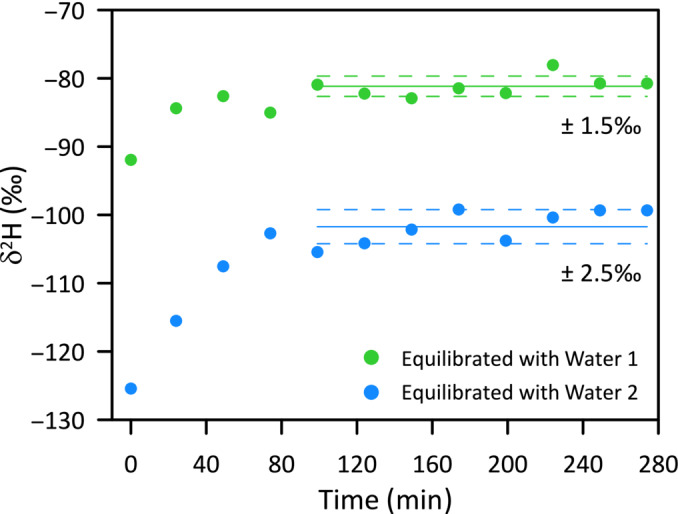
Measured δ^2^H (raw data) of a cellulose sample measured multiple times in an autosampler flushed with Argon with the setup “HTC triple.” Results of two runs with different equilibration waters are shown (δ^2^H_water1_ = −160‰ ± 1‰; δ^2^H_water2_ = −412‰ ± 1‰). Time = 0 indicates the analysis of the first sample.

One rather important influence on δ^2^H results was observed from the type of autosampler in use. The equilibrated samples may spend several hours in the autosampler, depending on their position in the tray and the length of the sample series. Therefore, they should not be in contact with atmospheric vapor, although equilibration should be slow at room temperature. We found the best option to be a so‐called zero‐blank autosampler, which has a cover with a seal. It can be evacuated using a vacuum‐pump and subsequently filled with helium. Samples in this autosampler therefore can be freed from significant traces of moisture that may have been condensed during (rapid) transport from the equilibration chamber to the autosampler. Furthermore, it is guaranteed that no moisture is in contact with samples during the entire analysis run. However, depending on manufacturer, this type of autosampler may not fit onto the HTC device, as is the case for the Pyrocube. This instrument has a heated (up to 80°C) autosampler tray but only a simple disk as a cover. We therefore constructed a hood that is connected to an Argon line and is used to flush the autosampler tray after samples have been placed as well as during the whole analysis. Nevertheless, we still found a certain degree of “equilibration loss” during initial 2 h for cellulose (see Figure 5) but not for sugars. We subsequently always applied a waiting time of 2 h to achieve a steady state before starting the isotope analysis of cellulose samples. During steady state, the calculated *x*_*e*_ was 0.07, whereas it was 0.13 for the first analysis in the example shown in Figure 5. But as samples and standards are treated in the same way, we found reliable results with this method (Figures 2 and 3), albeit with a lower calculated *x*_*e*_, lower also than obtained by HTC single analysis. We do not think that the lower *x*_*e*_ necessarily impacts the results as shown by interlaboratory comparison with the University of Basel, where 70°C equilibration temperature was applied and a relatively low value of *x*_*e*_ = 0.06 for cellulose [30] obtained. Nevertheless, a very strong correlation between δ^2^H_ne_ values obtained by the two laboratories was found (*r* ^2^ = 0.92 for leaf tissue and *r* ^2^ = 0.97 for wood tissue). The reason could be that the actual amount of freely exchangeable hydroxy H of cellulose is in this order of magnitude (5%) and much less than expected theoretically from the number of OH groups of cellulose (30%) [16, 29], similar as also found for samples equilibrated at room temperature [33]. When applying high vapor equilibration temperatures, intermolecular hydrogen bridges might break and therefore change the amount of exchangeable H^29^. For sugars, the waiting time of 2 h before starting the analysis proved not to be necessary, likely because equilibration of sugars with vapor at temperatures below 100°C is very slow due to their crystallization.

## 4 Concluding Remarks

Several considerations should be taken for a decision on individual or triple isotope analysis (Table 3). This should cover the whole process chain from sample type, vapor equilibration, and all aspects of instrument configuration up to postprocessing. A decision then can be made if triple isotope analysis is feasible. Although the triple analysis is very elegant in terms of efficiency and sample amount needed, some compromise is required in terms of precision. The highest precision for all three isotope ratios could mean up to four different analyses on three different systems, that is, 2× δ^2^H analysis with two waters on HTC‐single setup, one analysis for oxygen (HTC without equilibration), and one for carbon (EA combustion). This will multiply not only the time needed per sample but also the costs, considering machine operating time and technician hours (for weighing the samples in silver capsules and for running the equipment). Depending on the research question, sample type, and the number of samples to be analyzed, the triple isotope analysis can be the optimal choice, particularly when a large number of samples of the same type are to be analyzed (e.g., tree‐ring cellulose), which all are expected to have a similar *x*_*e*_. Reactor dimensions also play a role, as the tube in tube setup in some TC/EAs only allows few samples to be analyzed before a reactor change is required.

**TABLE 3:** Overview on instrumental factors, their influence on the isotope analysis, and considerations for single versus multiple isotope analysis.

Topic	Considerations	Impact on analysis
Sample type	Cellulose, sugars or organic material	High N‐content can be problematic
Standards	Type of material and isotope ranges	Similar material as sample if possible and wide range of isotope values for all analyzed isotope ratios
Vapor Equilibration	Equilibration temperature, 1 or 2 equilibrations	> 100°C for sugars needed; 2 waters/analysis for highest precision, 1 water for routine analysis of same sample type
Autosampler type	Zero blank, Ar flushing, simple cover	Equilibration loss possible
Reactor type	Glassy carbon or Chromium	Chromium best for nitrogen‐containing compounds, but only allows δ^2^H
Interface type	Differential dilution of H_2_ and CO	H_2_ sample signal is typically smaller than CO and should be adapted by changing the dilution within analysis
IRMS	Peak jump from H_2_ to CO	Necessary for triple isotope analysis
Precision	Tradeoff between precision and number of analyses	Highest for individual isotope analysis with two equilibrations
Postprocessing	Memory correction, calculation of non exchangeable H, two‐point calibration, quality control	Can be done in spreadsheet calculation, should follow a clear protocol for monitoring system performance

Triple isotope analysis may therefore mean, in practical considerations, that more samples can be analyzed to answer a certain research question. As the between sample isotope variation in biological samples is often large in comparison to the analytical precision, it may be better for estimating the true average to have more replicates with somewhat lower precision than a few samples with highest precision. For instance, tree δ^13^C values from the same forest stand can easily differ by 3‰ [34], which makes it important to have many replicates, but it matters less if these samples are measured at a precision of ± 0.1‰ or ± 0.2‰. This may be compared to the situation of atmospheric CO_2_, where for some questions, like long‐term atmospheric δ^13^C trends in clean air, utmost precision is required, also between laboratories [35], although such a precision is certainly not necessary for a keeling plot approach in a forest studying soil respiration [36]. Or as Leavitt [7] phrased it: “In the end, the isotopic results are only as good (representative, accurate and precise) as the natural variability of isotopes in trees and tree rings and sampling design allow.” Asking for the highest possible precision in all studies may preclude many of them.

By describing and promoting the triple isotope method as an elegant and efficient way, we hope to foster progress in many disciplines of stable isotope sciences. Providing details on the applied protocol, including pitfalls and drawbacks of the method, is essential for other laboratories to reproduce the results and reduce analytical differences between laboratories. This may hold, in particular, for hydrogen, where various protocols for vapor equilibration are in use and hardly any organic reference materials available with well‐defined δ^2^H values for carbon‐bound H. Using our straight‐forward method, more studies may potentially include this somewhat neglected isotope in future studies of environmental change.

## Author Contributions

**Matthias Saurer:** conceptualization, writing – original draft, writing – review and editing. **Manuela Oettli:** methodology, writing – review and editing. **Marco M. Lehmann:** conceptualization, writing – review and editing, methodology.

## Supporting information


**Figure S1** Inner structure of the equilibration chamber
**Figure S2** Outer structure of the equilibration chamber
**Figure S3** Zero‐blank autosampler on TC/EA
**Figure S4** Autosampler on Pyrocube with Plexiglas hood

## Data Availability

The data that support the findings of this study are available from the corresponding author upon reasonable request.
