# Supplementary material for: A Simple Method for Triple Stable Isotope Analysis of Cellulose, Sugar, and Bulk Organic Matter—Advances and Limitations
Source: Rapid Commun Mass Spectrom. 2024 Dec 2;39(4):e9957. doi: 10.1002/rcm.9957 (PMC11609901; doi:10.1002/rcm.9957)

Supplementary information for "A simple method for triple stable isotope analysis of cellulose, sugar and bulk organic matter – Advances and limitations", Rapid Communications in Mass Spectrometry

By Matthias Saurer, Manuela Oettli and Marco M. Lehmann

Photographs showing the equilibration device and autosamplers. See also Schuler et al. Plant Cell and Environment. 2022;45(1):12-22 for more details.

Figure S1

Inner structure of the equilibration chamber

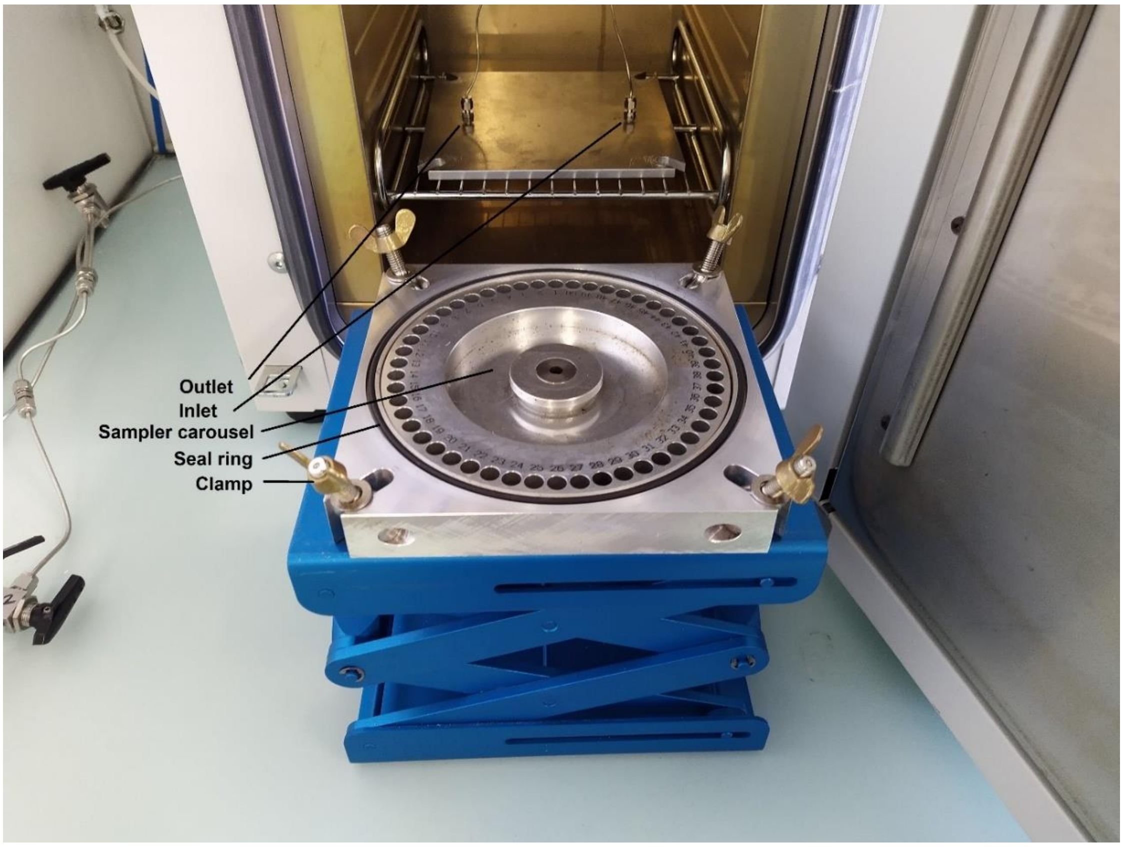

Figure S2

Outer structure of the equilibration chamber.

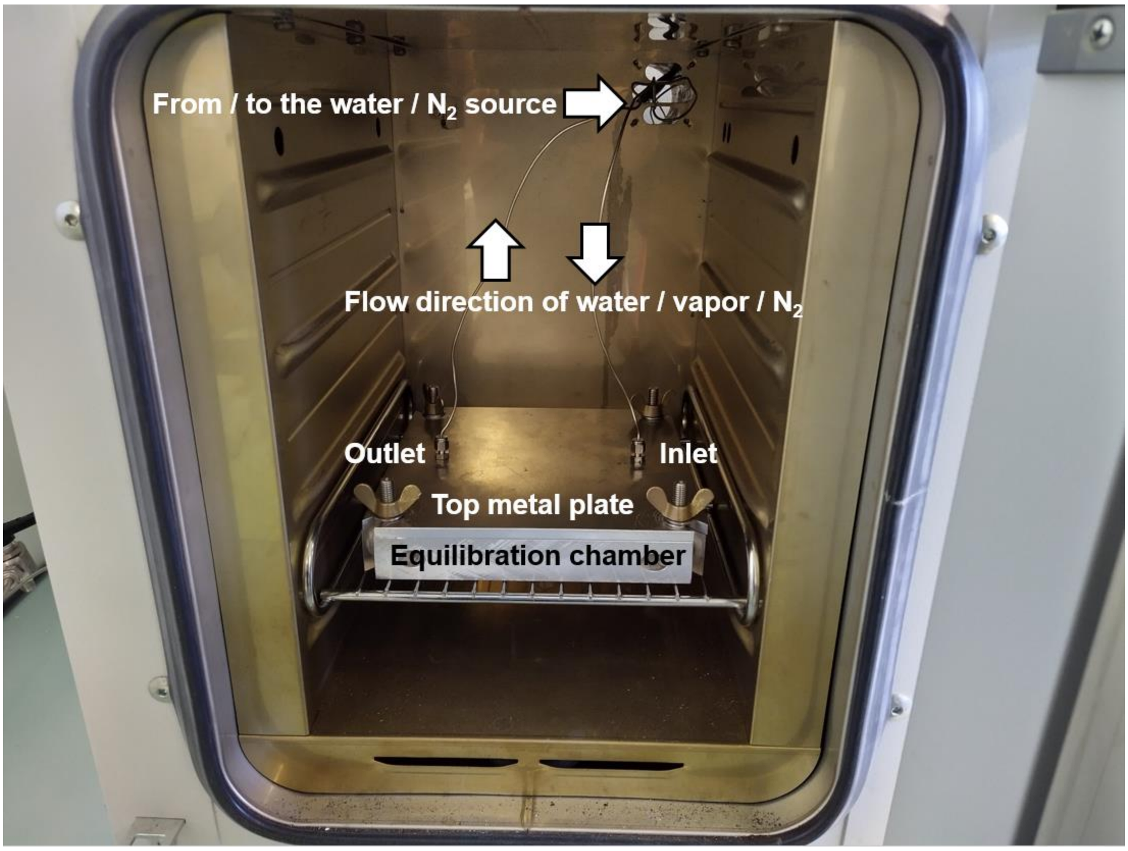

Figure S3

Zero-blank autosampler on TC/EA

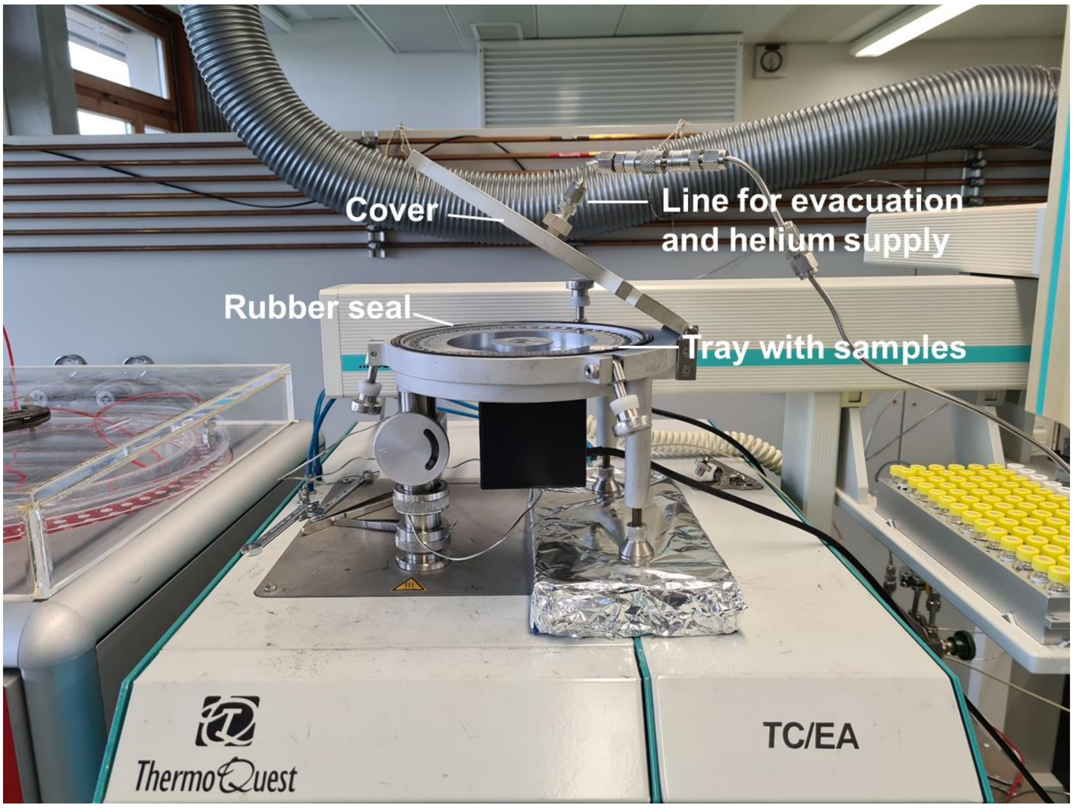

Figure S4

Autosampler on Pyrocube with Plexiglas hood

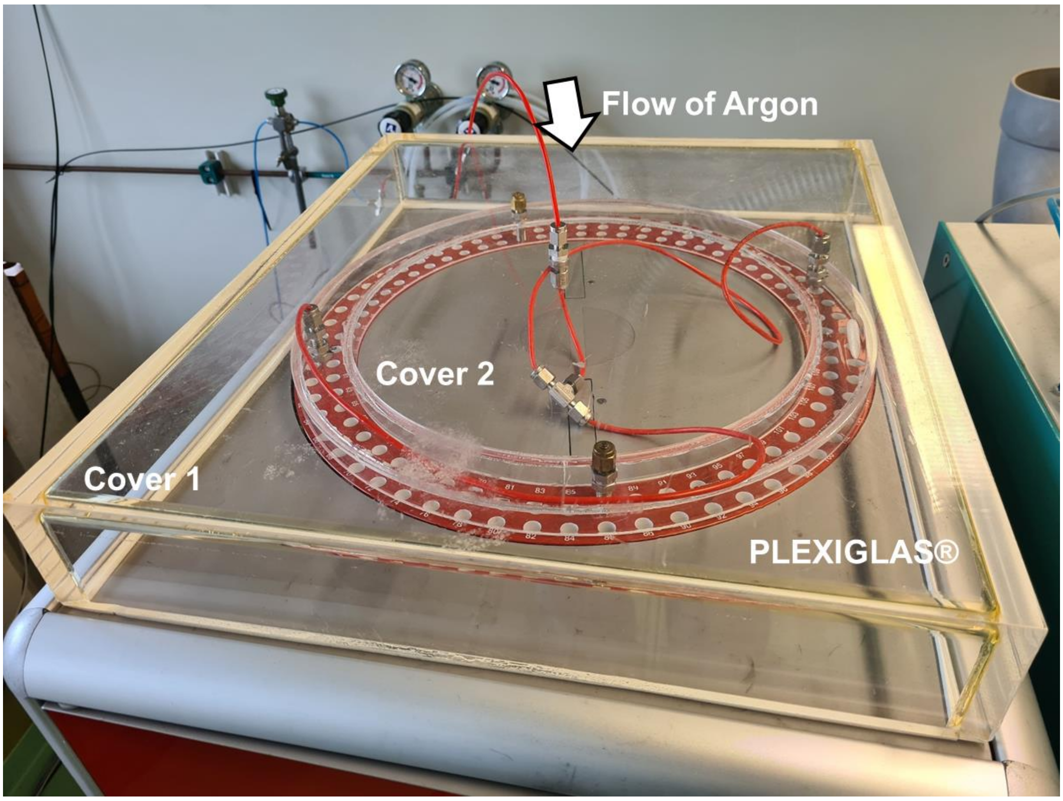

Supplement: Supplementary file 1 — Figure S1 Inner structure of the equilibration chamber Figure S2 Outer structure of the equilibration chamber Figure S3 Zero‐blank autosampler on TC/EA Figure S4 Autosampler on Pyrocube with Plexiglas hood [file RCM-39-e9957-s001.pdf]
